# Prevalence and antimicrobial susceptibility pattern of bacterial uropathogens among adult patients in Madinah, Saudi Arabia

**DOI:** 10.1186/s12879-023-08578-1

**Published:** 2023-09-06

**Authors:** Yahya A. Almutawif, Hamza M. A. Eid

**Affiliations:** Department of Medical Laboratories Technology, College of Applied Medical Sciences, Taibah University, Madinah, Saudi Arabia

**Keywords:** UTI, Prevalence, Bacteria, Uropathogens, Antimicrobial susceptibility test

## Abstract

**Background:**

Urinary tract infection (UTI) is considered one of the most prevalent infections that may lead to many renal complications. They account for almost 10% of all infections in Saudi Arabia, making them the second most common cause of emergency department admissions. Bacterial pathogens, primarily *Escherichia coli*, *Klebsiella* spp., *Enterococcus* spp., *Proteus* spp., and *Staphylococcus* spp. are the most causative agents of UTI. This study aims to evaluate the prevalence and antimicrobial susceptibility patterns of uropathogens in adult patients from Madinah, Saudi Arabia.

**Methods:**

A retrospective cross-sectional study was performed using data collected from patients who visited King Fahad General Hospital in Madinah, Saudi Arabia. Data included 16,803 urine bacterial cultures and their antimicrobial susceptibility profiles collected between January 2019 and October 2021.

**Results:**

Among the 16,803 tested samples, 3937 (23.4%) showed positive results for urine bacterial cultures. UTI prevalence was slightly higher in women (52.1%) than men (47.9%). *Escherichia coli* (29.8%) was the most prevalent, followed by *Klebsiella* spp. (23.2%) and *Pseudomonas* spp. (8.4%). As for Gram-positive bacteria, *Enterococcus* spp. (10.8%) were most common, followed by *Streptococcus* spp. (8%) and *Staphylococcus* spp. (3.3%). Gram-negative bacteria exhibited high resistance rates toward aztreonam (> 83.3%), ampicillin (78.8%), and cephalexin (68.5%). *Enterococcus* spp. displayed elevated resistance rates (> 62.3%) against ciprofloxacin, gentamicin, and tetracycline. Conversely, *Streptococcus* spp. showed substantial resistance rates (> 76.6%) toward colistin and trimethoprim/sulfamethoxazole.

**Conclusion:**

To optimize therapy and minimize the risk of multidrug-resistant uropathogenic infections, physicians should consider the local epidemiological trends and antimicrobial resistance patterns of prevalent uropathogens prior to initiating any empirical antibacterial therapy.

## Background

Urinary tract infection (UTI) is one of the most prevalent and serious infections worldwide, with more than 150 million new cases reported each year [1]. In Saudi Arabia, UTI represents almost 10% of all infection cases, which makes it the second most common cause of emergency admission [2]. It affects the urinary tract and prostate, causing significant complications and major health problems such as urinary tract dysfunction, bacterial septicemia, or even prominent kidney damage [3, 4].

UTI is an inflammation of the urinary tract caused by abnormal colonization of harmful microorganisms [5]. Normally, the urinary tract is free from any microorganisms, as it resists long-term colonization via different mechanisms. The protective mechanisms can be either mechanical (e.g., bladder emptying during micturition that washes off any residing microorganism) or physiological (e.g., host immunity, mucus production, and increased urea production) [6]. However, the inflammation disrupts the normal urinary tract function, which leads to incomplete microorganism clearance and eventually UTI development [6]. Nevertheless, acquiring the infection depends on several risk factors such as age, sex, period of hospitalization, pregnancy, diabetes, usage of urinary catheters, and genitourinary tract or immune system abnormalities [5, 7–9]. UTI can be either symptomatic (e.g., burning sensation during urination, fever, dysuria, and lower abdominal pain) or asymptomatic [5].

The type of UTI usually depends on the source of infection. For instance, community-acquired infection in healthy individuals is typically caused by rectal flora contamination via bacterial ascension to the urethra [10]. This type of infection is more common in women than in men owing to their genitourinary structures [11]. In contrast, nosocomial infection usually develops 48 h or later in hospitalized patients. Prosthetic devices such as urinary catheters also contribute to the occurrence of UTI in patients with nosocomial infection [12].

Several microorganisms are commonly involved in UTI, including bacteria, fungi, and viruses. However, bacteria are the most common causative agents for such an infection [1]. According to the literature and previous reports, Gram-negative bacteria account for nearly 90% of all UTI cases, while Gram-positive bacteria are responsible for only 10%. The most frequently identified uropathogen is *Escherichia coli*. However, other uropathogens, including *Klebsiella* spp., *Staphylococcus* spp., *Streptococcus* spp., *Proteus* spp., and *Pseudomonas* spp., may also be involved in UTI development [13–18].

The prevalence of uropathogens has been well established worldwide [5]. Nevertheless, the common uropathogen can be largely dependent on the geographical location. Each geographical region may exhibit a different pattern of uropathogens [19]. For example, more isolated regions or communities tend to share similar uropathogens when compared with multicultural or overlapped societies. Similarly, antimicrobial susceptibility patterns may vary among regions depending on public awareness and antibiotic usage [19]. This divergence underscores the challenge of handling uropathogens that exhibit resistance to important antibiotics, such as ß-lactams, aminoglycosides, polyketide, fluoroquinolones, sulfonamides and carbapenems [20–23]. This scenario places a substantial burden on healthcare, particularly when determining empirical therapy before urine culture outcomes are known. Consequently, comprehending the prevalence of UTI antimicrobial resistance becomes pivotal in guiding antibiotic choices for both empirical and precise therapeutic strategies.

In the current study, we investigated the prevalence of uropathogens in Madinah, Saudi Arabia. Madinah City is a well-known multicultural city that is visited by approximately 6–8 million Muslims from all over the world every year [24]. Thus, it is important to evaluate the prevalence and antimicrobial susceptibly trends of uropathogens, which could play a crucial role in determining the optimal empirical antibacterial therapy.

## Methods

### Sample collection and exclusion criteria

This retrospective cross-sectional study was performed using data collected from patients who visited King Fahad General Hospital in Madinah, Saudi Arabia, between January 2019 and October 2021. The collected data included both bacterial culture and antimicrobial susceptibility testing (AST) results from 16,803 patients suspected to have UTI. The study included all patients aged 18 years or older with suspected UTI. The urine sample culture was considered positive when bacterial counts exceeded 10^5^ CFU/mL, and the data from patients with positive results were included in the study. Meanwhile, all data collected from patients with urinary catheters or negative results (bacterial counts fewer than 10^5^ CFU/mL) were excluded.

### Bacterial culture, identification, and antimicrobial susceptibility testing

The samples were cultured on Cystine Lactose Electrolyte deficient (CLED) agar media (BD, USA) and incubated overnight at 37 °C. The bacterial isolates were initially identified based on the microbiology department protocol implemented in the hospital which included performing Gram staining and biochemical tests such as indole production, citrate utilization, urease test, and oxidase test for Gram-negative isolates while Gram-positive cocci were identified using catalase and coagulase tests. All isolates were also confirmed using different automated identification systems, including VITEK 2 (bioMérieux, USA) or Phoenix (BD, USA) chosen based on reagents availability. The AST was also performed using these systems according to the manufacturer’s protocol. This test covers up to 22 antibiotics including Amoxicillin + Clavulanic Acid, Amikacin, Ampicillin, Aztreonam, Ceftazidime, Cephalexin, Ciprofloxacin, Colistin, Cefuroxime, Cefazolin, Cefepime, Cefoxitin, Gentamicin, Imipenem, Levofloxacin, Meropenem, Nitroxoline, Trimethoprim/Sulfamethoxazole, Tigecycline, Piperacillin + Tazobactam for Gram-negative bacteria. While for Gram-positive bacteria the following antibiotics were used; Amoxicillin + Clavulanic Acid, Amikacin, Ampicillin, Aztreonam, Ceftazidime, Cephalexin, Ciprofloxacin, Colistin, Ceftriaxone, Cefotaxime, Cefuroxime, Cefazolin, Cefepime, Cefoxitin, Gentamicin, Imipenem, Levofloxacin, Meropenem, Nitroxoline, Norfloxacin, Trimethoprim/Sulfamethoxazole, Tigecycline, Piperacillin + Tazobactam, Penicillin, Erythromycin, Vancomycin, Oxacillin, High Gentamicin, Linezolid. The results were represented as sensitive, intermediate, or resistant and extracted automatically.

### Statistical analysis

Data were presented as numbers and percentages. All data were analyzed using GraphPad Prism v. 9.0 software (San Diego, USA).

## Results

### Number of positive cases and distribution of demographic data

Between January 2019 and October 2021, a total of 16,803 urine samples were sent for bacterial identification. These samples were collected from different hospital wards. A total of 3937 (23.4%) positive urine bacterial cultures were confirmed using VITEK 2 and Phoenix. The number of positive UTI cases was slightly higher in women (n = 2051; 52.1%) than in men (n = 1886; 47.9%) (Table 1). Saudi nationals showed the highest prevalence of positive cultures (n = 2959; 75.1%), while the remaining were non-Saudi patients (n = 978; 24.9%) (Table 1).

**Table 1:** Demographical characteristics of UTI-positive patients

Sex	Men n. (%)	1886 (47.9)	
	**Women n. (%)**	2051 (52.1)	
**Nationality**	**n. (%)**	**Nationality**	**n. (%)**
**Saudi Arabia**	2959 (75.2)	**Ethiopia**	12 (0.3)
**Pakistan**	152 (3.9)	**Tunisia**	10 (0.25)
**Mauritania**	96 (2.4)	**Algeria**	9 (0.23)
**Syria**	85 (2.2)	**Kuwait**	7 (0.18)
**Egypt**	83 (2.1)	**Mali**	7 (0.18)
**Sudan**	74 (1.9)	**Senegal**	7 (0.18)
**Afghanistan**	65 (1.7)	**Jordan**	6 (0.15)
**Yemen**	64 (1.6)	**Mali**	6 (0.15)
**Indonesia**	56 (1.4)	**Morocco**	5 (0.13)
**India**	55 (1.4)	**Somalia**	4 (0.1)
**Nigeria**	41 (1.04)	**Turkey**	4 (0.1)
**Palestine**	39 (1)	**Malaysia**	2 (0.05)
**Bangladesh**	29 (0.77)	**Cameroon**	2 (0.05)
**Chad**	22 (0.56)	**Brunei**	1 (0.03)
**Burma**	20 (0.51)	**Ivory coast**	1 (0.03)
**Philippine**	13 (0.33)	**Lebanon**	1 (0.03)

## Distribution of the etiological agents of UTI

**Fig. 1:**
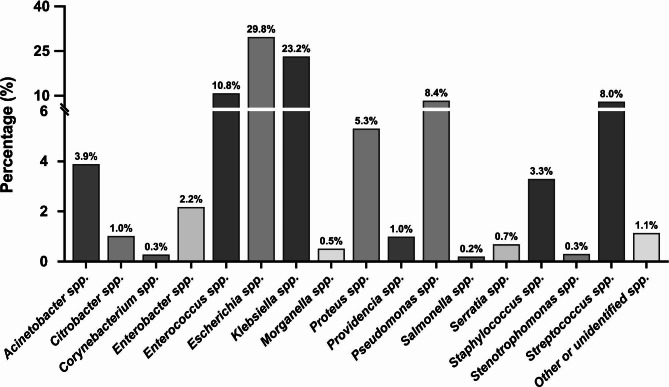
The overall identified bacterial genus isolated from UTI patients

Gram-negative bacteria were the most prevalent isolated group (n = 2998; 76.14%), while Gram-positive bacteria accounted for 22.7% of the cases (n = 894). The Enterobacterales family was the most frequently identified uropathogen (n = 2496; 63.4%). Among this family, *Escherichia* spp. were the most prevalent species (n = 1173; 29.8%), followed by *Klebsiella* spp. (n = 914; 23.2%). Meanwhile, *Enterococcus* spp. were the most prevalent Gram-positive bacteria (n = 426; 10.8%), followed by *Streptococcus* spp. (n = 315; 8%) (Fig. 1; Table 2).

**Table 2:** The overall identified bacterial species and their prevalence

*Genus*	n. *(%)*			*spp.*		n.		*spp.*		n.	
***Escherichia***	1173 (29.8)			*E. coli*		1172		*E. hermannii*		1	
***Klebsiella***	914 (23.2)			*K. oxytoca* *K. ozaenae*		12 10		*K. pneumoniae* *K. planticola*		890 2	
***Enterococcus***	426 (10.8)			*E. faecalis* *E. faecium*		301 111		*Other spp.*		14	
***Pseudomonas***	329 (8.4)			*P. aeruginosa* *P. acidovorans*		323 2		*P. putida* *Other spp.*		2 2	
***Streptococcus***	315 (8)			*S. agalactiae* *S. viridans* *S. pneumoniae*		269 16 12		*S. pyogenes* *Other spp.*		2 16	
***Proteus***	209 (5.3)			*P. mirabilis* *P. vulgaris*		193 8		*Other spp.*		8	
***Acinetobacter***	153 (3.9)			*A. baumannii* *A. lwoffii*		145 6		*A. haemolyticus*		2	
***Staphylococcus***	130 (3.3)			*S. aureus* *S. epidermidis* *S. haemolyticus*		103 9 7		*S. hominis* *S. saprophyticus* *Other Staph.*		3 1 7	
***Enterobacter***	86 (2.2)			*E. aerogenes* *E. agglomerans* *E. cancerogenus*		13 3 1		*E. cloacae* *Other spp.*		68 1	
***Citrobacter***	40 (1.0)			*C. braakii* *C. farmeri* *C. freundii*		4 5 11		*C.koseri* *C.sedlakii* *C. youngae*		16 1 3	
***Providencia***	39 (1.0)			*P. rettgeri*		23		*P. stuartii*		16	
***Serratia***	27 (0.7)			*S. fonticola* *S. liquefaciens*		4 1		*S. marcescens*		22	
***Morganella***	20 (0.5)			*M. morganii*		20					
***Stenotrophomonas***	12 (0.3)			*S. maltophilia*		12					
***Corynebacterium***	11 (0.3)			*C. amycolatum* *C. jeikeium* *Other spp.*		1 1 1		* C. diphtheriae* *C. striatum* *C. urealyticum*		5 1 2	
***Salmonella***	8 (0.2)			*S. enterica ss. enterica (Subgroup I)*		1		*Other spp.*		7	
**Other or unidentified**			45 (1.1)								
**Total**		**3937**									

**Fig. 2:**
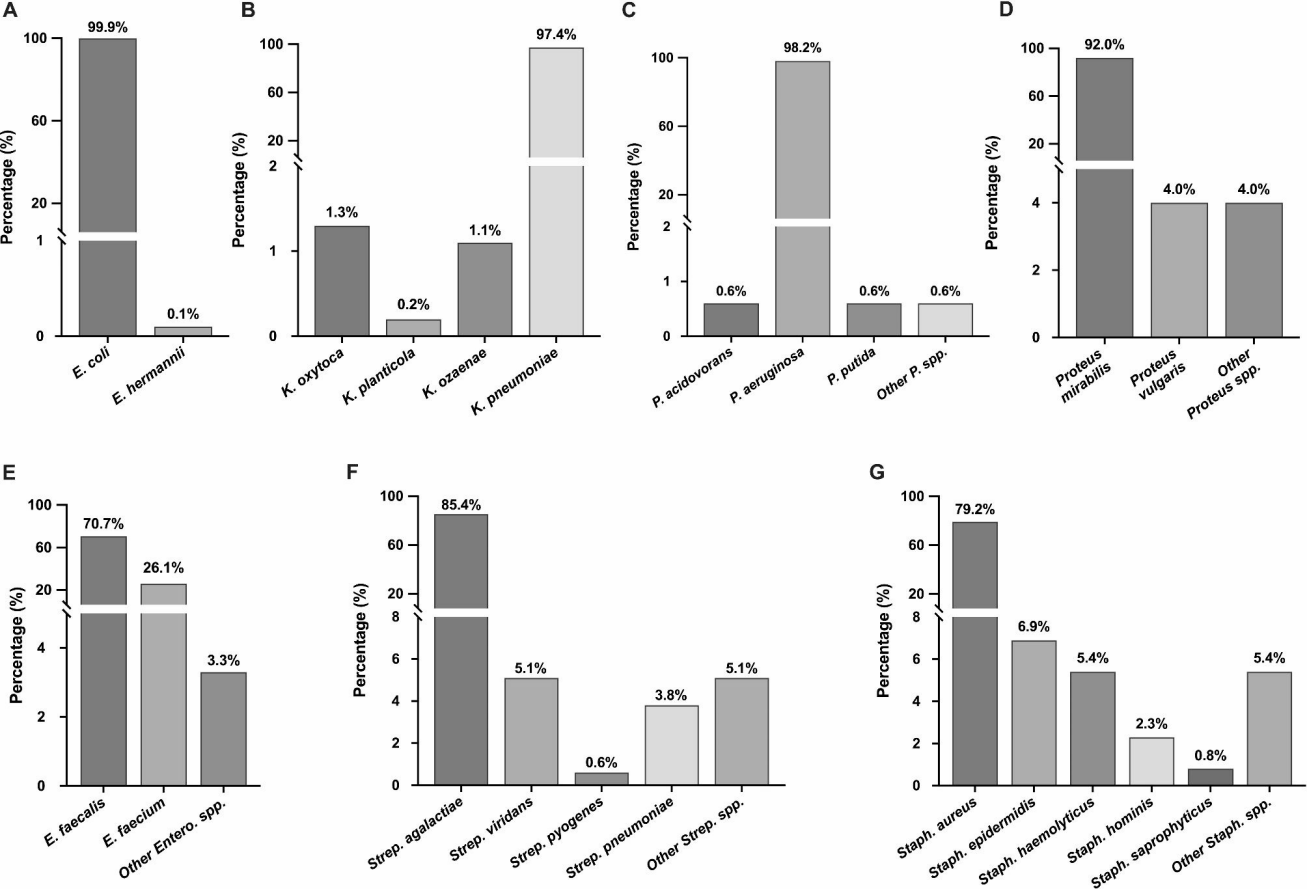
The predominant bacterial species isolated from UTI patients. **A**; *Escherichia* spp., **B**; *Klebsiella* spp., **C**; *Pseudomonas* spp., **D**; *Proteus* spp., **E**; *Enterococcus* spp., **F**; *Streptococcus* spp., and **G**; *Staphylococcus* spp

A total of 16 bacterial genera represented by 75 bacterial species were successfully isolated from the positive cultures (Fig. 1; Table 2). *E. coli* was the most predominant species within its genus (n = 1172; 99.9%) (Fig. 2A), while *Klebsiella pneumoniae* was the most commonly identified isolate among its genus (n = 890; 97.4%) (Fig. 2B). *Pseudomonas aeruginosa* and *Proteus mirabilis* were the most prevalent among their genera (n = 323; 98.2% and n = 193; 92%, respectively) (Fig. 2C and D). Meanwhile, *Enterococcus faecalis*, *Streptococcus agalactiae*, and *Staphylococcus aureus* were the most frequently identified isolates among their genera (n = 301; 70.7%, n = 269; 85.4%, and n = 103; 79.2%, respectively) (Fig. 2E–G).

### Prevalence of antimicrobial resistance among the identified uropathogens

**Fig. 3:**
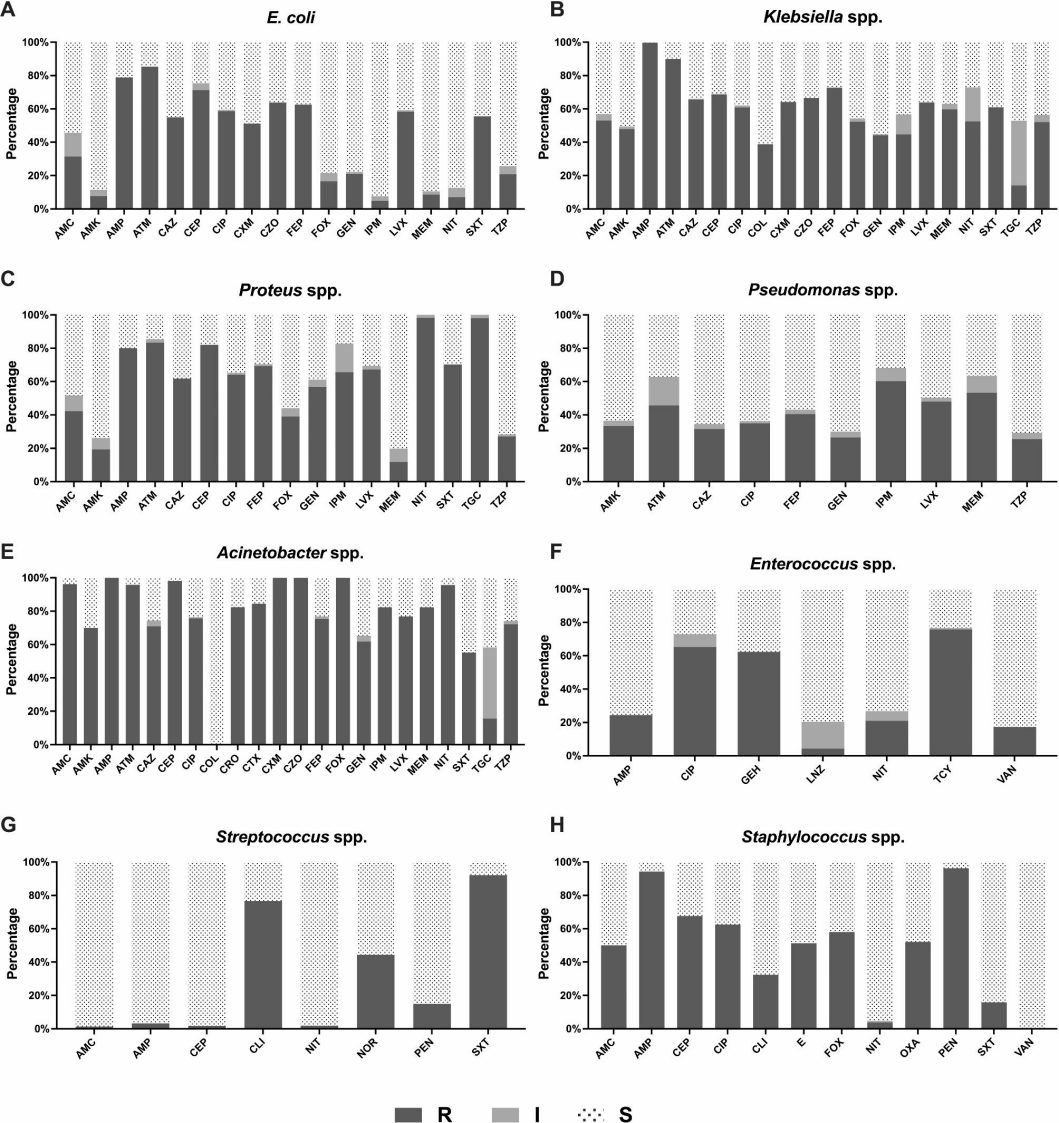
Antimicrobial sensitivity pattern of the most prevalent uropathogens. **A**; *Escherichia* spp., **B**; *Klebsiella* spp., **C**; *Proteus* spp., and **D**; *Pseudomonas* spp., **E**; *Acinetobacter* spp., **F**; *Enterococcus* spp., **G**; *Streptococcus* spp., and **H**; *Staphylococcus* spp. **R;** Resistant, **I;** Intermediate, **S;** Sensitive, **AMC;** Amoxicillin + Clavulanic acid, **AMK;** Amikacin, **AMP;** Ampicillin, **ATM;** Aztreonam, **CAZ;** Ceftazidime, **CEP;** Cephalexin, **CIP;** Ciprofloxacin, **COL;** Colistin, **CRO;** Ceftriaxone, **CLI;** Clindamycin, **CTX;** Cefotaxime, **CXM;** Cefuroxime, **CZO;** Cefazolin, **FEP;** Cefepime, **E;** Erythromycin, **FOX**; Cefoxitin, **GEH;** High Gentamicin, **GEN;** Gentamicin, **IPM;** Imipenem, **LNZ;** Linezolid, **LVX;** Levofloxacin, **MEM;** Meropenem, **NIT;** Nitroxoline, **NOR;** Norfloxacin, **OXA;** Oxacillin, **PEN;** Penicillin G, **SXT;** Trimethoprim/sulfamethoxazole, **TCY;** Tetracycline, **TGC;** Tigecycline, **TZP;** Piperacillin + tazobactam, **VAN;** Vancomycin

The AST data for the most prevalent bacterial genera/species *E. coli*, *Klebsiella* spp., *Proteus* spp., *Pseudomonas* spp., *Acinetobacter* spp., *Enterococcus* spp., *Streptococcus* spp., and *Staphylococcus* spp. were included (Fig. 3; Table 3). *E. coli* showed high resistance rates (> 50%) to ampicillin, aztreonam, cephalexin, ciprofloxacin, cefazolin, cefepime, levofloxacin, and trimethoprim/sulfamethoxazole. In contrast, *E. coli* was highly sensitive (> 88%) to imipenem, meropenem, and amikacin (Fig. 3A and Table 3).

**Table 3:** Antimicrobial sensitivity pattern of the most prevalent uropathogens

Antibiotic	*E. coli*				*Klebsiella spp.*				*Pseudomonas spp.*				*Proteus spp.*			
	#	R	I	S	#	R	I	S	#	R	I	S	#	R	I	S
Amoxicillin + Clavulanic Acid	772	243	110	419	615	326	24	265	*				116	49	11	56
Amikacin	635	49	24	562	628	301	10	317	186	62	6	118	145	28	10	107
Ampicillin	703	554	1	148	364	363	0	1	*				95	76	0	19
Aztreonam	419	357	1	61	407	366	0	41	94	43	16	35	96	80	2	14
Ceftazidime	978	535	4	439	805	528	3	274	310	98	9	203	186	115	0	71
Cephalexin	963	686	40	237	721	494	4	223	*				128	105	0	23
Ciprofloxacin	1012	593	8	411	790	481	9	300	300	105	4	191	181	116	2	63
Colistin	*				188	73	0	115	*				*			
Cefuroxime	241	123	1	117	195	125	0	70	*				*			
Cefazolin	239	152	1	86	194	129	0	65	*				*			
Cefepime	801	500	3	298	700	508	2	190	232	94	6	132	163	113	2	48
Cefoxitin	615	102	30	483	587	307	11	269	*				100	39	5	56
Gentamicin	928	195	10	723	791	350	5	436	287	76	10	201	185	105	8	72
Imipenem	598	29	17	552	584	261	70	253	176	106	14	56	93	61	16	16
Levofloxacin	587	342	5	240	524	334	2	188	177	85	4	88	134	90	3	41
Meropenem	436	37	9	390	511	305	17	189	150	80	15	55	152	18	12	122
Nitroxoline	1052	75	58	919	773	406	158	209	*				115	113	2	0
Trimethoprim/Sulfamethoxazole	1068	593	1	474	843	513	1	329	*				191	134	0	57
Tigecycline	*				320	45	124	151	*				51	50	1	0
Piperacillin + Tazobactam	793	165	38	590	737	384	32	321	290	74	11	205	166	45	2	119
**Antibiotic**	***Acinetobacter spp.***				***Enterococcus spp.***				***Streptococcus spp.***				***Staphylococcus spp.***			
	**#**	**R**	**I**	**S**	**#**	**R**	**I**	**S**	**#**	**R**	**I**	**S**	**#**	**R**	**I**	**S**
Amoxicillin + Clavulanic Acid	51	49	0	2	*				76	1	0	75	46	23	0	23
Amikacin	113	79	0	34	*				*				*			
Ampicillin	41	41	0	0	402	98	0	304	285	9	0	276	86	81	0	5
Aztreonam	72	69	0	3	*				*				*			
Ceftazidime	148	105	5	38	*				*				*			
Cephalexin	51	50	0	1	*				231	4	0	227	34	23	0	11
Ciprofloxacin	148	112	1	35	282	184	22	76	*				48	30	0	18
Colistin	76	0	0	76	*				64	49	0	15	37	12	0	25
Ceftriaxone	34	28	0	6	*				*				*			
Cefotaxime	32	27	0	5	*				*				*			
Cefuroxime	31	31	0	0	*				*				*			
Cefazolin	31	31	0	0	*				*				*			
Cefepime	138	104	2	32	*				*				*			
Cefoxitin	42	42	0	0	*				*				50	29	0	21
Gentamicin	144	89	5	50	*				*				*			
Imipenem	124	102	0	22	*				*				*			
Levofloxacin	125	96	0	29	*				*				*			
Meropenem	124	102	0	22	*				*				*			
Nitroxoline	46	44	0	2	388	81	23	284	219	4	0	215	106	4	1	101
Norfloxacin	*				*				79	35	0	44	*			
Trimethoprim/Sulfamethoxazole	143	79	0	64	*				189	174	0	15	114	18	0	96
Tigecycline	96	15	41	40	120	91	1	28	*				*			
Piperacillin + Tazobactam	140	101	3	36	*				*				*			
Penicillin	*				*				114	17	0	97	52	50	0	2
Erythromycin	*				*				*				41	21	0	20
Vancomycin	*				283	49	0	234	*				65	0	0	65
Oxacillin	*				*				*				96	50	0	46
High Gentamicin	*				215	134	0	81	*				*			
Linezolid	*				93	4	15	74	*				*			

* Not performed

*Klebsiella* spp., including the most isolated *K. pneumoniae*, showed high resistance rates (≥ 60%) to most of the tested antibiotics. In particular, the resistance rates to ampicillin and aztreonam were > 89%. In contrast, *Klebsiella* spp. demonstrated high sensitivity rates (> 55%) to colistin and gentamicin (Fig. 3B; Table 3). A similar pattern of antibiotic resistance (resistance rate of approximately ≥ 50%) was seen in *Proteus* spp., but the sensitivity rate to meropenem was relatively high (80.3%). In contrast to *Klebsiella* spp., *Proteus* spp. showed high resistance rates (98%) to nitroxoline and tigecycline (Fig. 3C; Table 3). Amikacin and meropenem were the most effective antibiotics against *Proteus* spp. at sensitivity rates of 80.3% and 73.8%, respectively. *Pseudomonas* spp. were almost 50% sensitive to 7 of 10 antibiotics and 60.2% resistant to imipenem (Fig. 3D; Table 3). In contrast, *Acinetobacter* spp. showed the highest resistance rates (> 55%) among all identified bacteria; colistin was the only effective antibiotic at a sensitivity rate of 100% (Fig. 3E; Table 3).

Among the Gram-positive bacteria, *Enterococcus* spp. were the most predominantly identified genera in 426 samples (10.8%). They demonstrated sensitivity rates of > 73% to ampicillin, linezolid, nitroxoline, and vancomycin (Fig. 3F; Table 3) and resistance rates of > 62% to ciprofloxacin, gentamicin, and tetracycline. *Streptococcus* spp. and *Staphylococcus* spp. were isolated from 315 (8%) and 130 (3.3%) samples, respectively. *Streptococcus* spp. showed sensitivity rates of > 85% to Augmentin, ampicillin, cephalexin, and penicillin, while *Staphylococcus* spp. demonstrated resistance rates of ≥ 50% to the same antibiotics (Fig. 3G and H; Table 3). Meanwhile, *Staphylococcus* spp. showed higher sensitivity rates to colistin and trimethoprim/sulfamethoxazole than did *Streptococcus* spp. However, nitroxoline was effective against both genera at a resistance rate of < 3.8%.

## Discussion

This retrospective study was conducted to determine the prevalence, etiology, and antimicrobial susceptibility patterns of uropathogens isolated from patients who visited King Fahad General Hospital in Madinah, Saudi Arabia, between January 2019 and October 2021.

Herein, nearly one-quarter of all culture samples were positive for certain bacteria. The prevalence of positive cultures in our study was 23.4%, consistent with that in other studies conducted in Saudi Arabia and Iraq [25, 26]. In Hai’l, Saudi Arabia, and Baghdad and Erbil, Iraq, the prevalence has been demonstrated to be 19.6% [25] and 26.58% and 22%, respectively [26]. However, conflicting findings regarding the prevalence of UTI have also been reported [27, 28]. For instance, a high prevalence rate (32.3%) was documented in a study conducted at different hospitals in Uganda [5]. Another study conducted in Italy reported that 541 of 1745 (31%) urine samples showed positive bacterial cultures [29]. Meanwhile, lower prevalence rates (< 9.8%) have been reported in India, Bangladesh, Ethiopia, and Peru [6, 30–32]. These discrepancies could be attributed to the geographical distribution where the studies were conducted as well as the sample size, hygienic practices, awareness, educational level, community customs and traditions, and sex [26].

Regarding sex, women had a higher prevalence of UTI (52.1%) than men (47.9%). This finding is consistent with most previous reports [5, 6, 31, 32]. Several studies have proposed factors that could increase the prevalence of UTI among certain patients, including the proximity of the urethra to the anus and less acidic pH of the vaginal surface in women, wider and shorter urethra, sexual behavior, incontinence, and poor hygienic practices [11, 33, 34].

The samples collected from Saudi patients with suspected UTI showed the highest prevalence of positive cultures (74.2%), which could be attributed to the higher proportion of Saudis than that of non-Saudis in this study. Conversely, other nationalities with large communities in Madinah such as Pakistanis and Mauritanians demonstrated the highest prevalence among the non-Saudis (Table 1).

The Gram-negative bacteria were the most prevalent isolates from our patients’ urine samples. The Enterobacterales family was the predominant bacterial family. *E. coli* was the most prevalent isolated bacterial species (n = 1172; 29.8%), followed by *K. pneumoniae* (n = 889; 22.4%) and *P. aeruginosa* (n = 323; 5.1%) (Table 2). Despite the multicultural nature of Madinah, our findings concerning the most predominant Gram-negative bacteria are consistent with several reports in Saudi Arabia and elsewhere [5, 6, 25–27, 31, 32, 35].

Among the Gram-positive isolates, *E. faecalis* was the most frequently identified species (n = 301; 7.6%), followed by *S. agalactiae* (n = 269; 6.8%). Consistent with our findings, several studies, including a study conducted in Sakaka, Saudi Arabia, reported *Enterococcus* spp. as the most commonly isolated Gram-positive uropathogen [31, 35, 36]. However, other uropathogens such as *S. aureus*, *S. agalactiae*, and coagulase-negative Staphylococcus have also been reported as the most frequently identified Gram-positive bacteria [25, 37]. These discrepancies could be attributed to the methodology implemented in the data collection, sensitivity of bacteria identification systems, or differences in the inclusion criteria or sample size. Nevertheless, other factors, including hygienic practices, awareness, and educational level within the studied community, may also contribute to the etiological variations, which must be considered in future studies [26].

In the AST, *E. coli* showed > 50% resistance to 10 of 18 tested antibiotics. It exhibited the highest resistance rates to aztreonam, ampicillin, and cephalexin at 85.2%, 78.8%, and 71.2%, respectively (Table 3). These findings agree with other reports of resistance rates between 70% and 90% against these antibiotics [31, 36, 38]. On the contrary, *E. coli* was highly sensitive to imipenem, meropenem, and amikacin at the rates of 92.3%, 89.4%, and 88.5%, respectively. The high sensitivity rates in this study are closely similar to those in the previous work by Rahman et al. (97.89%, 80.87%, and 88.65%, respectively) [31] (Table 3).

*Klebsiella* spp. *has* a resistance pattern that is relatively similar to that of *E. coli* although with a higher resistance rate (Table 3). Notably, *Klebsiella* spp. demonstrated 59.7% and 44.7% resistance rates to meropenem and imipenem, respectively, compared with *E. coli*. Moreover, the resistance to imipenem occurred at a much faster rate owing to the higher intermediate resistance level. These findings contradict other reports within Saudi Arabia that imipenem and meropenem are still effective against *Klebsiella* spp. at resistance rates of < 24%. Colistin has been shown to be effective against *Klebsiella* spp. at a resistance rate of 8.3%; in this study, a much higher resistance rate (38.8%) was observed [35].

For *Proteus* spp., a previous study has shown meropenem as the most effective antibiotic at a sensitivity rate of 100% and nitroxoline as the least effective antibiotic at a resistance rate of 80% [37]. Our study showed increased resistance rates for both antibiotics (Fig. 3C; Table 3). Nevertheless, the same previous study has reported high sensitivity rates to meropenem and imipenem (90%), in contrast to our sensitivity rates of 60.2% and 53.3%, respectively (Fig. 3C; Table 3). Herein, *Acinetobacter* spp. showed high resistance rates to almost all tested antibiotics, except for colistin, which showed a 100% sensitivity rate (Fig. 3E; Table 3), similar to that reported in Northern Saudi Arabia [35].

*Enterococcus* spp. showed a 17.3% resistance rate to vancomycin and a 4.3% resistance rate to linezolid (Fig. 3F; Table 3). Similarly, Taher et al. reported that *Enterococcus* spp. had a resistance rate of 13% to vancomycin and 7.5% to linezolid [35]. In addition, Rahman et al. reported a sensitivity rate of 94.05% and 79.76% to vancomycin and linezolid, respectively [31]. *Staphylococcus* spp. were most highly sensitive to vancomycin (100%), followed by nitroxoline (95.3%). However, they showed high resistance rates of > 51% to ampicillin, erythromycin, and oxacillin, consistent with previously reported data [31, 36]. *Streptococcus* spp. showed a high resistance rate to trimethoprim/sulfamethoxazole (92.1%) which is markedly higher than the 25% resistance rate that had been reported in Hai’l, Saudi Arabia [25].

A notable limitation of this study is the localized scope, which does not fully capture the broader population trends or account for potential UTI patterns. Moreover, although the findings are valuable, their relevance to the entirety of Saudi Arabia might be limited due to the country’s substantial geographical expanse and diverse environments. In addition, the challenge of antibiotic shortages posed a significant obstacle, given the vital importance of thoroughly investigating the antibiotic sensitivity patterns of all isolated bacteria. Thus, the availability of antibiotics is essential to provide a comprehensive idea about the antibiogram in a specific community. Furthermore, it would be valuable to expand the scope of the study findings by incorporating an examination of UTI prevalence and antimicrobial patterns within specific risk groups and different hospitals.

## Conclusions

Incorrect practices performed by healthcare practitioners, including erroneous prescriptions or over-prescription of antibiotics, are considered a main contributor to developing and spreading bacterial resistance. Physicians usually follow general guidelines in treating patients with UTIs. Thus, the local epidemiological trends and antimicrobial sensitivity rates of common bacteria are typically neglected. In this retrospective study, we focused on the prevalence, etiology, and antimicrobial sensitivity trends of uropathogens in a local setting. This study could provide insights into a successful antimicrobial selection for UTI treatment. We strongly suggest that any empirical antibiotic selection should consider the local epidemiological trends and resistance patterns of the most common uropathogens rather than implementing a universal guideline. The findings could also serve as a basis for implementing new policies to control the emergence of multidrug-resistant uropathogens.

## Data Availability

The data are available upon request in accordance with confidentiality and privacy regulations from the corresponding author.

## List of Abbreviations

UTI: Urinary tract infection
AST: Antimicrobial susceptibility test
